# A Novel Approach to Standard Techniques in the Assessment and Quantification of the Interventricular Systolic Relationship

**DOI:** 10.1186/1476-7120-9-42

**Published:** 2011-12-20

**Authors:** Steven R Bruhl, Mangeet Chahal, Samer J Khouri

**Affiliations:** 1 Division of Cardiovascular Medicine, University of Toledo Medical Center, Toledo, Ohio, USA

**Keywords:** ventricular interdependence, ventricular function, TAPSE, MAPSE, tissue Doppler

## Abstract

**Background:**

Blood flow between the right and left ventricles is subject to the continuity equation and systolic ventricular interdependence. Quantification of this relationship might aid in understanding inter-ventricular function. The purpose of this study was to evaluate and quantify ventricular interdependence by directly comparing right and left ventricular systolic function though echocardiographic surrogates of right and left ventricular systolic function such as MAPSE, TAPSE, RV TVI and LV TVI.

**Methods:**

This study prospectively evaluated 51 healthy participants (mean age, 41 ± 17 years) by resting echocardiography. In addition to standard measurements, tricuspid annular plane of systolic excursion, (TAPSE), mitral annular plane of systolic excursion (MAPSE), and the peak annulus systolic velocity of the right ventricular (RVs) and left ventricular (LVs) free walls were measured by M-mode and pulsed wave Doppler tissue echocardiography and further evaluated for variance across age, gender, and body surface area.

**Results:**

TAPSE (22.1 ± 2.9 mm) was over 54.5% greater than MAPSE (14.3 ± 2.6 mm) and RVs was 64.4% greater than LVs. The LV to RV systolic relationship measured by MAPSE/TAPSE and LVs/RVs ratios were 0.66 ± 0.14 and 0.76 ± 0.21 respectively. These values were not significantly affected by age, gender or body surface area (BSA).

**Conclusion:**

MAPSE/TAPSE and LVs/RVs ratios appear stable across age, gender, and BSA potentially making them good surrogates of systolic ventricular relationship and interdependence.

## Background

The differences between the right and left ventricle go far beyond differences in shape and thicknesses and further include differences in cardiac myofibril concentration and orientation leading to complex differences in the way each ventricle ejects blood [1]. However, despite these differences, according to the continuity equation, flow in one area must equal flow in a second area provided no shunts are present. In cardiology, this principle is most frequently applied to the calculation of valve areas, but since this principle is derived from the conservation of mass law, it also applies to blood flow between the right and left ventricles. When one or both ventricles fail, this delicate balance is upset leading to the shunting of blood manifested by systemic and/or pulmonary edema. Since the difference between balance and imbalance can be the difference between health and significant morbidity and mortality, we hypothesis that this interventricular relationship could be quantified using simple echocardiographic techniques. This knowledge could further provide insight into the balance between normal right and left ventricular function and quantify pathological systolic imbalance between the two ventricles. In order to accomplish this we sought simple and accurate echocardiographic techniques to assess right and left ventricular function independently in order to then directly compare systolic performance of each ventricle to the other.

Although there are many surrogates of LV systolic function, the complex shape of the right ventricle (RV) makes its' functional assessments much more challenging. Despite newer techniques such as three-dimensional echocardiography and cardiac magnetic resonance, the cost and expertise required to utilize these modalities currently make them impractical for both screening and follow up evaluation [2]. However, M-mode derived tricuspid annular motion, also known as tricuspid annular plane systolic excursion (TAPSE) is a simple and widely used method for echocardiographic assessment of RV systolic function [3-7]. Furthermore, TAPSE has been validated as a good surrogate of RV function in multiple MRI and 3D echocardiographic studies including a recent MRI study by Dirk et al which showed a good correlation between TAPSE and RV ejection fraction (r = 0.52; p < 0.001) [8,9].

This previous MRI study by Dirk et al as well as a study by Qin et al [10] revealed that the vertical motion of the mitral valve, known as the mitral annular plane of systolic excursion (MAPSE), also correlates well with LV systolic function [8,11].

Similarly, measurements of peak mitral and tricuspid valvular velocities using pulsed wave Doppler tissue imaging (DTI) have also been shown in multiple studies to correlate well with ejection fraction[3,6,12,13]. This correlation remained consistent regardless of both pulmonary artery pressure^6 ^and even poor image resolution [14].

Based on the simplicity and reproducibility of these surrogate markers of systolic function, we chose the longitudinal systolic motion of the lateral walls of both ventricles as measured by TAPSE and MAPSE as well as the peak systolic annulus velocity (S) of the same segments as measured by Doppler Tissue Imaging (DTI).

## Methods

After Institutional Review Board approval, data was collected on fifty one healthy adult volunteers without any known history of cardiac disease using two-dimensional and Doppler echocardiography at our non-invasive imaging laboratory. All echocardiograms were done according to the recommendations and standards of the American Society of Echocardiography using a standard commercially available ultrasound systems (Vivid 7, S6 and Vivid i; GE Healthcare, Milwaukee, WI), and stored digitally for subsequent analysis using software (EchoPac, Version 3.1, GE). Participants were excluded from the study if they had a rhythm other than sinus rhythm, a history of pulmonary hypertension, atrial fibrillation, complete right or left bundle branch block, pacemakers, defibrillators or more than mild valvular disease. Participants with a history of heart failure, myocardial infarction, previous cardiac surgery, an LVEF < 55%, a left atrial index > 28 cc/m^2^, LV or RV hypertrophy, LV or RV enlargement, or more than mild valvular heart disease were also excluded.

Baseline variables collected included age, BSA, gender, LVEF, left ventricular interventricular septal thickness, left atrial volume index, and right ventricular systolic pressure (RVSP). Eligible participants were then divided by age into 3 groups; group I < 30, group II 30-50, and Group III > 50 years old.

**Figure 1:**
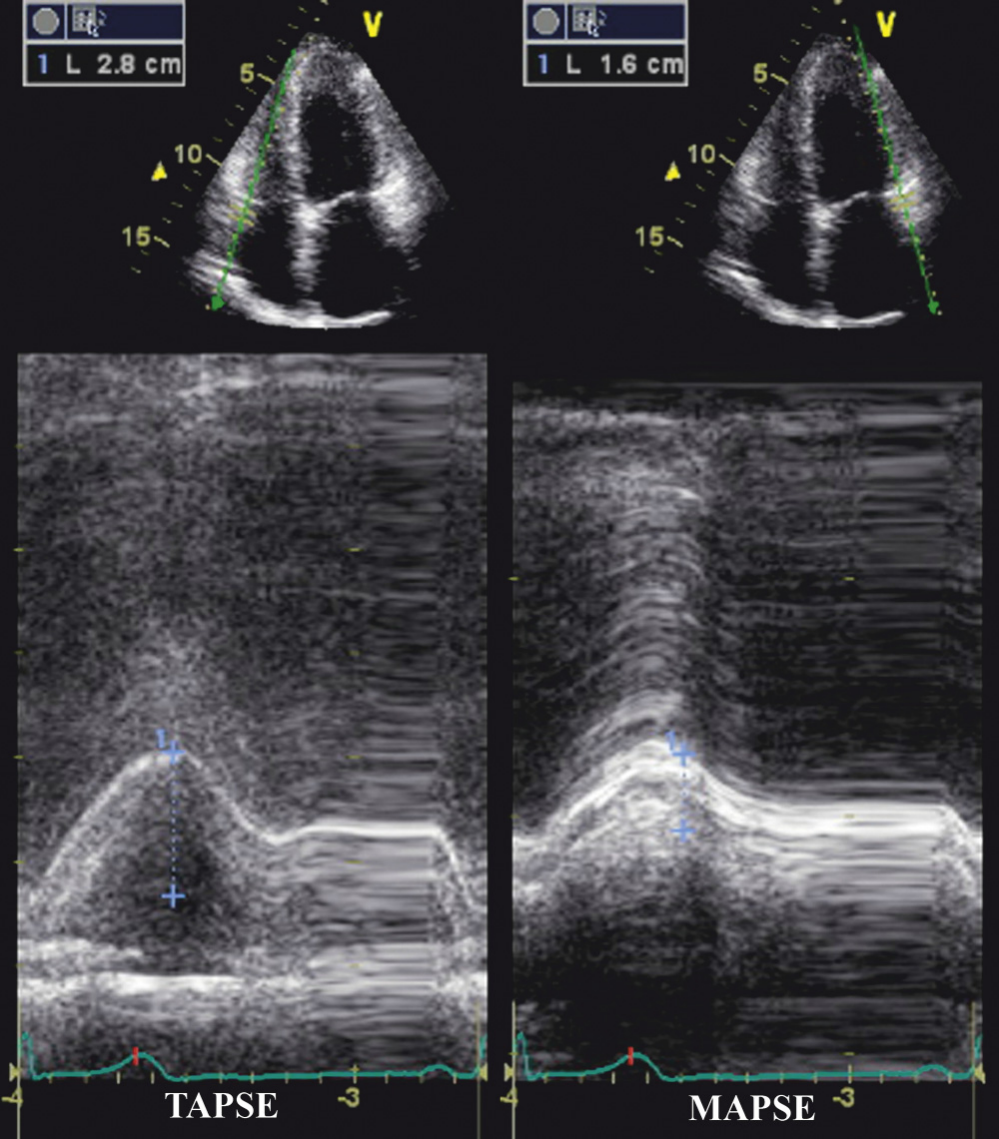
**Examples of M-mode derived tricuspid annular plane of systolic excursion (TAPSE) (left), and mitral annular plane of systolic excursion (MAPSE) (right) from the four chamber view**.

**Figure 2:**
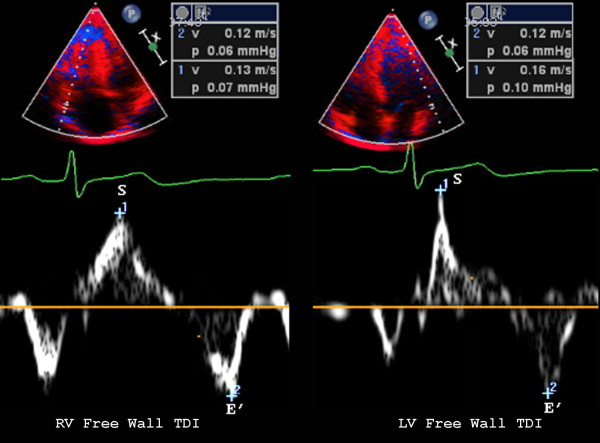
**Examples of color Doppler tissue imaging lateral to the tricuspid valve (left) and mitral valve (right) from the four chamber view including peak systolic (S) and diastolic (E') velocities**.

From the four chamber view, TAPSE and MAPSE (Figure 1) were measured from the lateral aspect of each annulus using M-mode imaging. Similarly, peak systolic tissue Doppler velocities were measured from the basal RV free wall just lateral to the mitral and tricuspid valves (Figure 2) using fixed 5-mm sample volumes. Baseline demographic and clinical characteristics for continuous variables were expressed as mean ± SD and tested for differences using the Student's t test or χ^2 ^when appropriate. The study population was also divided into 3 groups by age including those < 30 years, 30-50 years, and > 50 years old as well as by sex. Analysis of variance (ANOVA) was performed in order to compare multiple echo measurements across age groups. Age and BSA dependencies of MAPSE/TAPSE and LVs/RVs measurements were evaluated by linear regression analysis. An intra and interobserver agreement analysis using the Kappa statistic was performed to determine consistency among evaluators. For all measurements, a two-sided *P *value of less than 0.05 was considered statistically significant. All statistical analysis was performed using SPSS for Windows, version 17.0, SPSS Inc, Chicago, Illinois, USA) and GraphPad Prism version 5.0 (La Jolla, CA).

## Results

Basic demographic and echocardiographic data were collected and were separated by gender. All variables were similar between the two groups except for an increased BSA among male subjects (Table 1).

**Table 1:** Demographics and basic echocardiographic data from 51 healthy Individuals. BSA, body surface area

	Female N = 29	Male N = 21	P value
**Age, y**	42.5 ± 16.1	39.7 ± 17.7	0.744
**BSA, m**^**2**^	1.8 ± 0.2	1.9 ± 0.2	0.02
**LA volume index, cc/m**^**2**^	14.9 ± 10.6	14.4 ± 6	0.824
**LVIVS, cm**	0.9 ± 0.2	0.9 ± 0.1	0.823
**RVSP, MMHg**	28.8 ± 4.2	27.7 ± 4.4	0.919
**LVEF, %**	56.2 ± 4.2	55 ± 2.8	0.811

LVIS, left ventricular interventricular septum; RVSP, right ventricular systolic pressure; LVEF, left ventricular ejection fraction

On average, TAPSE (22.1 ± 2.9 mm) was over 54.5% greater than MAPSE (14.3 ± 2.6 mm) and when MAPSE was directly compared to TAPSE, the resultant MAPSE/TAPSE systolic interventricular ratio was 0.66 ± 0.14. When these values were further analyzed for variance, this relationship was remarkably consistent regardless of age (*P *= 0.51), sex (*P *= 0.19), or BSA (*P *= 0.78) (Table 2).

**Table 2:** Measured echocardiographic variables by age and gender. MAPSE, mitral annular plane of systolic excursion

	< 30y	30-50	> 50	ANOVA	Female	Male	
	N = 16	N = 18	N = 17	P value	N = 29	N = 22	P value
**Peak annular excursion, mm**							
**Tricuspid (TAPSE)**	22.2 ±.9	22.6 ± 2.8	21.8 ± 3.2	0.70	22 ± 2.8	22.5 ± 3.2	0.52
**Mitral (MAPSE)**	14.3 ± 2	15.1 ± 2.9	13.6 ± 2.7	0.25	14.6 ± 2.8	14 ± 2.2	0.40
**MAPSE/TAPSE**	0.65 ± 0.12	0.68 ± 0.15	0.64 ± 0.13	0.64	0.68 ± 0.16	0.63 ± 0.09	0.19

**Peak s-wave velocity, cm/s**							
**RV Free Wall (S**_**RV**_**)**	13.4 ± 2.2	12.2 ± 2	13.4 ± 2.8	0.20	13.2 ± 2.2	12.6 ± 2.7	0.38
**LV Free Wall (S**_**LV**_**)**	10.5 ± 2.8	9 ± 2.1	10 ± 2.7	0.64	10 ± 2.5	9.6 ± 2.7	0.61
**LVs/RVs**	0.78 ±0.21	0.75 ± 0.19	0.76 ± 0.2	0.82	0.76 ± .18	0.77 ± 0.22	0.80

TAPSE, tricuspid annular plane of systolic excursion RV, right ventricle; LV, ventricle; ANOVA, analysis of variance.

**Figure 3:**
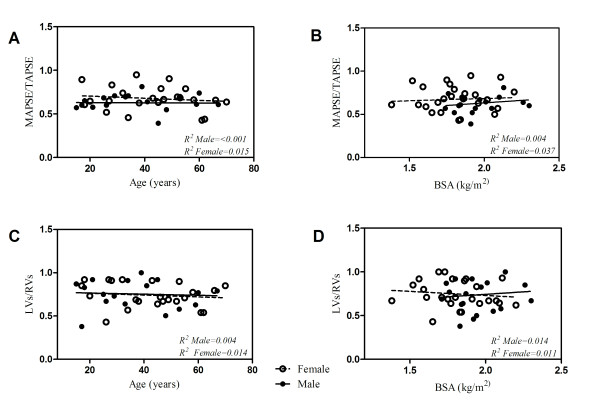
**Relationship of TAPSE/MAPSE to age (A) and body surface area (BSA) (B)**. Relationship of S_LV_/S_RV _to age (C) and BSA (D).

This same process was applied using Doppler tissue imaging of the RV and LV lateral free walls and revealed that RVs was on average 64.4% greater than LVs with a LVs/RVs systolic interventricular ratio of 0.76 ± 0.19. Likewise, no significant difference was seen when these values were analyzed for variance across age (*P *= 0.24), BSA (*P *= 0.70) and gender (*P *= 0.80) (Table 2). Linear regression analysis of MAPSE/TAPSE and LVs/RVs ratios were first separated by gender and then graphed against age and BSA. The resultant linear regression lines were both flat and almost identical regardless of gender further confirming that age, BSA, and gender had no significant effect on MAPSE/TAPSE or S_LV_/S_RV _ratios (Figure 3). The average Kappa coefficient for inter and intraobserver agreement for the measured values were 0.659 (p <.0.001) and 0.814 (p < 0.001) respectively.

## Discussion

The results of this study revealed several interesting qualities of right and left systolic relationship. First, the surrogates of RV function, TAPSE and the velocity of the RV free wall were almost always greater than their LV counterparts. On average TAPSE was over 54.4% greater than MAPSE, and RVs was over 64.4% greater than LVs. Although the etiology of this can only be speculated, the ability of the RV to sustain this increased basal to apical movement and tissue velocity despite its significantly reduced muscle mass is likely related to the reduced resistance of the pulmonary vascular circuit as compared to the LV systemic circuit. A second likely factor is that although a large portion of RV contraction occurs in the basal to apical plane, a large portion of left ventricular contraction also occurs in other planes. This fact was illustrated by Carlsson et al which showed that although 80% of RV stroke volume results from shortening in the longitudinal plane, this motion accounts for only 60% of left ventricular total stroke volume.^1^

A second finding is that the measurements of TAPSE (2.1 cm ± 0.35), MAPSE (1.4 cm ± 0.26), RVs (13.02 cm/s ± 2.4) and LVs (9.76 cm/s ± 2.58) fell within relatively tight dispersions across the population. Furthermore, when each of the LV surrogates was directly compared to its respective RV surrogate, the resultant systolic interventricular ratios remained tightly distributed (TAPSE/MAPSE = 0.66 ± 0.14 and S_LV_/S_RV _= 0.76 ± 0.19) regardless of age, BSA or gender. Although these results were somewhat unexpected, the reason for this might be suggested by a separate study by Carrlson et al. which showed that despite the different contributions of longitudinal motion toward RV and LV total stroke volume, the actual percentage of total stroke volume contributed by each ventricle remained relatively consistent, even when compared between healthy adults, athletes, and patients with dilated cardiomyopathy with reduced LVEF [15].

## Limitations

Although this study was relatively small and only included healthy subjects, the consistency of TAPSE/MAPSE and LVs/RVs ratios make them unique and potentially good instantaneous or perhaps even good chronological measurements of right and left ventricular systolic interdependence. Furthermore, if this relationship remains consistent in larger studies, this technique could potentially be used to diagnose and evaluate patients with diseases that might preferentially alter either right or left ventricular function. The most classic example would be in the setting of constrictive pericarditis, but could also include acute pulmonary emboli or pulmonary hypertension where RV function might be affected more than LV function leading to a smaller MAPSE/TAPSE ratio. Conversely, disease processes that preferentially affect left ventricle function such as aortic stenosis or acute left ventricular infarcts might be expected to lead to a more positive MAPSE/TAPSE or LVs/RVs ratio. In these conditions, the ratios could not only help detect subtle changes in the interventricular systolic relationship, but might also be used to monitor progression or improvements that result from specific treatments.

The goal of this pilot study was to assess the systolic relationship and ventricular relationship and interdependence of the right and left ventricle and to assess the feasibility and reproducibility of directly comparing ventricular systolic function using echocardiographic surrogate markers as well as establish normal interventricular baseline values. One limitation intrinsic to all pilot studies is its relatively small sample size as well as the fact that it only contained normal subjects. Although we admit these shortcomings, we also believe that the goals of our study were met and thus a larger prospective study which will include patients with isolated RV and LV diseases is currently underway.

## Conclusion

Both MAPSE/TAPSE and LVs/RVs ratios are reproducible, easy to obtain, and appears stable across age, BSA, and gender potentially making them good surrogates for RV and LV systolic interdependence. Although these findings require further validation in a larger study, these measurements might aid in the diagnosis or management of patients in which interventricular systolic function is altered by acute or chronic processes.

## Patient Consent

This is a retrospective study. The Internal Review Board (IRB) granted an exemption to review the data in echocardiograms retrospectively.

## Competing interests

No authors associated with the writing of this manuscript have conflicts of interest to report.

## Authors' contributions

All the authors contributed equally to this manuscript and have read and approved the final manuscript.

## References

[B1] Carlsson M Ugander M Heiberg E The quantitative relationship between longitudinal and radial function in left, right, and total heart pumping in humans Am J Physiol Heart Circ Physiol 2007 293 H636 644 10.1152/ajpheart.01376.2006 17307988

[B2] Nijveldt R Germans T McCann GP Semi-quantitative assessment of right ventricular function in comparison to a 3D volumetric approach: a cardiovascular magnetic resonance study Eur Radiol 2008 18 2399 2405 10.1007/s00330-008-1017-7 18523785

[B3] Alam M Wardell J Andersson E Characteristics of mitral and tricuspid annular velocities determined by pulsed wave Doppler tissue imaging in healthy subjects J Am Soc Echocardiogr 1999 12 618 628 10.1053/je.1999.v12.a99246 10441217

[B4] Kjaergaard J Sogaard P Hassager C Quantitative echocardiographic analysis of the right ventricle in healthy individuals J Am Soc Echocardiogr 2006 19 1365 1372 10.1016/j.echo.2006.05.012 17098140

[B5] Koestenberger M Ravekes W Everett AD Right ventricular function in infants, children and adolescents: reference values of the tricuspid annular plane systolic excursion (TAPSE) in 640 healthy patients and calculation of z score values J Am Soc Echocardiogr 2009 22 715 719 10.1016/j.echo.2009.03.026 19423286

[B6] Saxena N Rajagopalan N Edelman K Tricuspid annular systolic velocity: a useful measurement in determining right ventricular systolic function regardless of pulmonary artery pressures Echocardiography 2006 23 750 755 10.1111/j.1540-8175.2006.00305.x 16999693

[B7] Tamborini G Pepi M Galli CA Feasibility and accuracy of a routine echocardiographic assessment of right ventricular function Int J Cardiol 2007 115 86 89 10.1016/j.ijcard.2006.01.017 16750277

[B8] Dirk L Henning S Stephanie L 1113 MAPSE and TAPSE measured by MRI correlate with left and right ventricular ejection fraction and NTproBNP in patients with in dilated cardiomyopathy Journal of Cardiovascular Magnetic Resonance 2008 10 A238 10.1186/1532-429X-10-S1-A238

[B9] Forfia PR Fisher MR Mathai SC Tricuspid annular displacement predicts survival in pulmonary hypertension Am J Respir Crit Care Med 2006 174 1034 1041 10.1164/rccm.200604-547OC 16888289

[B10] Qin JX Shiota T Tsujino H Mitral annular motion as a surrogate for left ventricular ejection fraction: real-time three-dimensional echocardiography and magnetic resonance imaging studies Eur J Echocardiogr 2004 5 407 415 10.1016/j.euje.2004.03.002 15556815

[B11] Elnoamany MF Abdelhameed AK Mitral annular motion as a surrogate for left ventricular function: correlation with brain natriuretic peptide levels Eur J Echocardiogr 2006 7 187 198 10.1016/j.euje.2005.05.005 16046188

[B12] Alam M Hoglund C Thorstrand C Longitudinal systolic shortening of the left ventricle: an echocardiographic study in subjects with and without preserved global function Clin Physiol 1992 12 443 452 10.1111/j.1475-097X.1992.tb00348.x 1505166

[B13] Meluzin J Spinarova L Bakala J Pulsed Doppler tissue imaging of the velocity of tricuspid annular systolic motion; a new, rapid, and non-invasive method of evaluating right ventricular systolic function Eur Heart J 2001 22 340 348 10.1053/euhj.2000.2296 11161953

[B14] Yuda S Inaba Y Fujii S Assessment of left ventricular ejection fraction using long-axis systolic function is independent of image quality: a study of tissue Doppler imaging and m-mode echocardiography Echocardiography 2006 23 846 852 10.1111/j.1540-8175.2006.00331.x 17069603

[B15] Carlsson M Ugander M Mosen H Atrioventricular plane displacement is the major contributor to left ventricular pumping in healthy adults, athletes, and patients with dilated cardiomyopathy Am J Physiol Heart Circ Physiol 2007 292 H1452 1459 17098822 10.1152/ajpheart.01148.2006

